# Polymer Brush Coating and Adhesion Technology at Scale

**DOI:** 10.3390/polym12071475

**Published:** 2020-06-30

**Authors:** Kristian Birk Buhl, Asger Holm Agergaard, Mie Lillethorup, Jakob Pagh Nikolajsen, Steen Uttrup Pedersen, Kim Daasbjerg

**Affiliations:** 1 Interdisciplinary Nanoscience Center (iNANO), Aarhus University, Gustav Wieds Vej 14, DK 8000 Aarhus C, Denmark; kbb@inano.au.dk (K.B.B.); aha@inano.au.dk (A.H.A.); jakob@radisurf.com (J.P.N.); 2 Radisurf ApS, Arresoevej 5B, DK-8240 Risskov, Denmark; 3 Department of Chemistry, Aarhus University, Langelandsgade 140, DK-8000 Aarhus C, Denmark

**Keywords:** polymer brushes, adhesion, industrial application, entanglement, ATRP at scale

## Abstract

Creating strong joints between dissimilar materials for high-performance hybrid products places high demands on modern adhesives. Traditionally, adhesion relies on the compatibility between surfaces, often requiring the use of primers and thick bonding layers to achieve stable joints. The coatings of polymer brushes enable the compatibilization of material surfaces through precise control over surface chemistry, facilitating strong adhesion through a nanometer-thin layer. Here, we give a detailed account of our research on adhesion promoted by polymer brushes along with examples from industrial applications. We discuss two fundamentally different adhesive mechanisms of polymer brushes, namely (1) physical bonding via entanglement and (2) chemical bonding. The former mechanism is demonstrated by e.g., the strong bonding between poly(methyl methacrylate) (PMMA) brush coated stainless steel and bulk PMMA, while the latter is shown by e.g., the improved adhesion between silicone and titanium substrates, functionalized by a hydrosilane-modified poly(hydroxyethyl methacrylate) (PHEMA) brush. This review establishes that the clever design of polymer brushes can facilitate strong bonding between metals and various polymer materials or compatibilize fillers or nanoparticles with otherwise incompatible polymeric matrices. To realize the full potential of polymer brush functionalized materials, we discuss the progress in the synthesis of polymer brushes under ambient and scalable industrial conditions, and present recent developments in atom transfer radical polymerization for the large-scale production of brush-modified materials.

## 1. Introduction

Most products today are hybrids manufactured by combining a number of materials to optimize functionality, performance, and price. A key challenge is the formation of strong joints, often between dissimilar materials. Mechanical interlocking with bolts, screws, and flanges is still used in many products permitting disassembly for repair or recycling, but the method is relatively expensive, bulky, impractical, and often not a choice. The use of natural adhesive substances, such as starch glue, has been exploited for joining natural materials such as wood and paper. However, strong and reliable adhesives, for joining e.g., metal and plastic parts, remain an exciting challenge to the surface chemist because several parameters and properties must be considered. First, the chemical compatibility between the two material surfaces is crucial, enabling the formation of strong attractive physical forces or even chemical bonds. Second, the formed interactions, physical or chemical, should be mechanically strong and stable, and differences in the thermal expansion coefficient of the two materials should be considered. Third, on-demand disassembly will in many cases be a requirement for the next generation of adhesives, well aligned with the vision for a circular economy. 

Adhesives are typically polymers dissolved in a given solvent system and applied as a thin (μm) layer between materials that need to be joined. For many adhesives, certain curing times and temperatures are required for solvents to evaporate and/or the adhesive to form chemical crosslinks before the maximum strength of the joint is achieved. Using classical adhesives, incompatibilities when bonding dissimilar materials, thick adhesive layers, leachable substances escaping from the joint, and the irreversible assembly of the joint are some of the challenges persisting in applications. 

Polymer brushes are polymer chains chemically linked in one end to a substrate surface. A polymer brush coating used for adhesion is typically <100 nm in thickness resulting in extremely low material consumption, low risk of chemical leaching, and high precision in the dimensions of the joint. Polymer brushes represent an exciting new opportunity to achieve precise control of surface chemistry, enhance material compatibility, and allow the introduction of reactive functional groups to enable strong chemical bonding. For almost 30 years, polymer brushes have been one of the most popular research areas in organic nano-architecture and thousands of papers have been published covering everything from detailed mechanistic studies on the polymerization techniques to promising applications [1,2]. Polymer brushes have received much attention due to the versatile and broad scope of applications such as stimuli-responsive surfaces [3,4], cell-adhesion [5,6], wetting [7], anti-fouling [8], energy storage [9], and heterogeneous catalysis [10,11]. 

Polymer brushes present a flexible approach to give surfaces a pseudo-3D spatial arrangement of functional groups with nanoscale control over both film thickness and grafting density. A range of surface-initiated (SI) polymerization techniques used for polymer brush synthesis are described in the literature. They include free radical polymerization [12,13], ring-opening metathesis polymerization [14], nitroxide-mediated polymerization [15], and photoiniferter-mediated polymerization [16], The most widely used techniques to fabricate well defined polymer brushes are reversible addition-fragmentation chain transfer (RAFT) polymerization and SI-atom transfer radical polymerization (SI-ATRP) [17,18]. Recent advances in RAFT- and ATRP-related reactions have improved the oxygen tolerance and lowered the polymerization times and catalyst loadings, making these techniques highly promising from an industrial point of view. Beyond RAFT- and ATRP-related processes, photo-polymerizations have recently been developed to tolerate atmospheric levels of oxygen. The polymerizations are metal-free, which in terms of environmental and health considerations is an attractive trait [19,20]. However, being the most popular and matured polymer brush synthesis method, we herein put our focus on ATRP-related processes and their upscaling. 

Polymer brushes obtained via SI-ATRP are made in a two-step process consisting of (1) anchoring of initiator sites to a surface and (2) controlled polymerization initiated from these radical generating moieties. Typically, organosilane or organophosphate chemistry is used to anchor initiators to a wide range of inorganic oxide materials such as silica, alumina, and stainless steel [21,22]. Likewise, initiators based on aryl diazonium salts have been covalently bonded by electrografting to metals and carbon materials [23,24]. Thiol- and disulfide-based self-assembled monolayers (SAMs) have commonly been used to make initiator-functionalized noble metal substrates, such as from gold, platinum, and copper [17]. As a result, the introduction of surface-attached ATRP initiators is highly substrate dependent. Some methods require several synthetic steps while others are simple one-step modifications. Since this topic is well described in the literature, it will not be covered in detail here [2,23,24]. 

The significant development in the ATRP methodology has led to a huge library of monomers available for polymer brush synthesis, including a large variety of methacrylates, acrylates, methacrylamides, acrylamides, and styrenic, pyridinic, and vinylic monomers [25,26,27], Furthermore, post-polymerization reactions have extended the library into branched subsections of chemical designs, making polymer brushes applicable for numerous applications [11,27]. The diversity of monomers available for polymer brush synthesis is important for achieving both compatibility and adhesion, as the surface properties of the polymer brush functionalized material can be tailored to imitate bulk polymer materials, including thermosets, thermoplastics, and elastomers. Adhesion is obtained either via physical interactions where the polymer brushes unify with the bulk polymer material through interpenetration and entanglement, or via chemical crosslinks where reactive groups in the polymer brush react and form covalent bonds to the bulk polymer melt. The result is an ultra-strong and -tight molecular adhesive, enabling the bonding of dissimilar, incompatible materials such as metal, metal oxides, and carbon materials to a broad variety of polymers. The polymer brush adhesion zone only constitutes a 30−100 nm interphase, and polymer brush-based joints find applications spanning sectors from medico and sensors to offshore and electronics. Using polymer brushes to make different materials compatible can be utilized for nanocomposite synthesis as well. In fact, polymer brush functionalization of fillers, such as carbon materials [28], SiO_2_ [29], CeO_2_ [30], and TiO_2_ nanoparticles (NPs) [31] are known to facilitate efficient mixing with otherwise incompatible polymeric matrices [32]. The technique is highly promising for the production of new hybrid materials, as it prevents agglomeration and phase separation of fillers. The same strategy has been used in our work over the last decade to improve the compatibility between two surfaces of dissimilar materials to form strong and durable bonds.

In this paper, we intend to expand the vast library of applications related to polymer brushes by giving a thorough description of polymer brushes as a novel joining technology for strong and durable bonding of dissimilar materials. We present an overview of the technology by giving an account of our own work in the field, combined with an industrial perspective. We bolster the review by relating our work on adhesion to the use of polymer brushes as compatibilizers for NP fillers, and by including theoretical and computational considerations on the topic. Finally, we set the stage for transitioning the technology from research laboratories to modern production lines, by commenting on industrially relevant methods for the upscaling of SI-ATRP to larger objects and for developing tolerant methods for synthesizing polymer brushes under ambient conditions.

## 2. Physical and Chemical Interactions in Interphases between Polymer Brushes and Bulk Polymer Matrices

**Figure 1:**
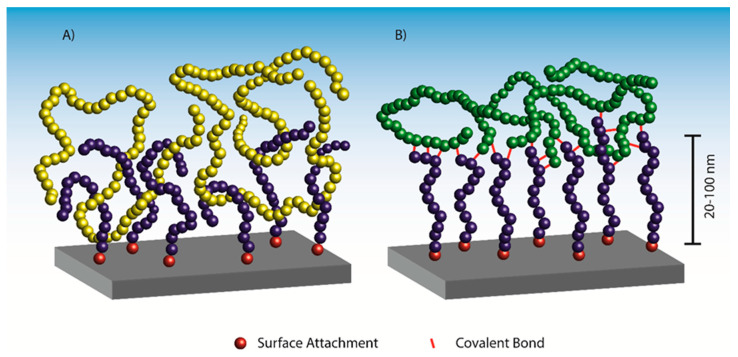
Sketch of polymer brush-based adhesion via (**A**) the entanglement and physical interactions and (**B**) the covalent bonds between the polymer brushes and a bulk polymer matrix.

Adhesion may originate from physical interactions such as Van der Waals, electrostatic or dipole–dipole interactions, or from covalent bonds or weaker hydrogen bonds. While commercial adhesives, e.g., epoxy, polyurethane, and acrylic adhesives utilize both chemical and physical bonds, the ability of polymer brushes to interpenetrate into bulk polymer matrices enables a unique mode of interaction in terms of chain entanglement. Entanglement constitutes an important interaction in the category of non-covalent bonds, resulting in a nanoscale mechanical interlocking (Figure 1A). While entropy generally drives mixing, the enthalpy of mixing is of key importance when considering polymers. The enthalpy of mixing between polymer chains increases with chain length, while the entropy of mixing remains constant for each polymer chain regardless of length. This makes enthalpy the main factor determining whether polymer brushes will entangle with a bulk polymer or not. For this reason, entanglement is only expected when the enthalpy of mixing is negative or zero, e.g., between miscible or identical polymer types. A similar behavior is observed for polymer brushes either swelling into an extended brush conformation in good solvents, or collapsing into a mushroom-like structure in poor solvents [33,34]. The compatibility and miscibility signify the importance of having access to a large library of monomers for the synthesis of polymer brushes [35].

Studies have shown that the chain ends of polymer brushes in the dry state are folding back and are buried inside the brush layer due to self-entanglement [36]. This was investigated by Ruths et al. using a surface force apparatus [37]. Reduced stretching of the brushes after compression for one hour was ascribed to the formation of self-entanglements during compression. Spiliopoulos et al. utilized neutron reflection on a deuterated brush system to directly probe the distribution of non-deuterated chain ends throughout a dense polystyrene (PS) brush [38]. They found that chain ends are present in the entirety of the brush structure, indicating strong self-entanglement in the brush [38]. Another study used electromechanical interferometry to investigate the elastic properties of high-density poly(methyl methacrylate) (PMMA) brushes which also proved that stretched brushes had a considerable amount of self-entanglement [39]. If self-entanglement can happen in a dense brush configuration, it is not surprising that similar entanglement may occur between polymer brushes and a dissolved or melted bulk polymer matrix, providing the foundation for adhesion. 

According to computational studies, the chain length (*N*) and grafting density (Σ) of polymer brushes have a nonmonotonic impact on the adhesive strength to a bulk polymer due to the phase behavior of polymer brushes [40]. Depending on Σ, the polymer brush morphology is divided into two regions: (1) for low Σ chains are isolated and do not overlap but (2) as Σ increases, chains start to overlap and stretch to reduce excluded volume interactions. In the first region, the work of adhesion increases with Σ while for the second, the dense polymer chains start to phase separate (interpenetration of polymer chains decreases), resulting in a decrease in the work of adhesion. Thus, from a theoretical point of view, there is an optimum polymer brush-grafting density regime favoring interpenetration and entanglement. Whether or not high grafting densities disfavor entanglement in practical applications remains to be determined, but experimental indications of a stagnation or even a decrease in adhesive performance with very dense brushes have been observed [41]. Once the entanglements are formed, they result in very strong bonding, as the pull-out of brush chains from the bulk polymer is disfavored entropically and by virtue of the excluded volume interactions between entangled polymer brushes and chains in the adhering bulk polymer. 

If a polymer brush compatible with the adherend cannot be directly synthesized, polymer interpenetration and entanglement is unachievable. In that case, polymer brushes with reactive handles (functional groups) may be exploited to form covalent bonds between the brush and the adherend, thus facilitating adhesion through covalent bond formation (Figure 1B). This approach often utilizes the curing of the adherend, e.g., in thermosets or rubbers, to facilitate bond formation to the polymer brushes and thus obtain adhesion. In some cases, a post-polymerization modification of the polymer brushes is necessary to introduce the reactive handles, because the presence of these functional groups in the monomer is incompatible with the ATRP reaction. Poly(glycidyl methacrylate) (PGMA) has been utilized for post-polymerization modifications in a wide array of systems, because the epoxide groups are compatible with the ATRP process and react readily with nucleophiles such as thiols [42], polysulfides [43], azides [36], and amines [44]. Functional groups introduced in the polymer brush in the post-polymerization modification may react and form covalent bonds to the adherend e.g., at elevated temperatures or with pressure in a molding process.

## 3. Adhesion Based on Polymer Brushes

Shimizu et al. first demonstrated the use of polymer brushes as an adhesive technology utilizing the concept of entanglement [41]. They grew PMMA brushes from stainless steel (SS) substrates and subsequently achieved entanglement and bonding by injection overmolding with bulk PMMA (Table 1). Different grafting densities of PMMA brushes were synthesized by varying the surface density of the initiator. The latter was controlled via the reaction time for an acylation step, used to synthesize the surface initiator for the ATRP step. After overmolding with bulk PMMA, the strength of adhesion was measured by tensile tests showing that low grafting densities indeed resulted in a lower bonding strength, compared to the higher densities of the PMMA brushes. While the work of adhesion increased with density initially, a slight lowering was observed at the maximal grafting density. The molding time was also varied, and at least 300 s at 120 °C was needed to achieve the maximal bond strength, facilitated by the necessary interpenetration of brushes into the bulk polymer. These findings are well in line with simulations showing that mixing time has a large impact on the establishment of entanglement [35,45]. At intermediate grafting density and a molding time of 300 s, a tensile strength of 8 ± 2 MPa was obtained. The most significant result was the failure mode after tensile testing, where thick PMMA residues from the bulk PMMA were present on the SS surfaces due to cohesive failure in the bulk polymer. From an industrial perspective, these results are highly promising since polymer brushes of only ~40 nm in dry film thickness result in an adhesive strength prevailing the strength of the bulk material near the interface. 

The work of Shimizu et al. represents the simplest case where the surface chemistry of a metal substrate is tailored to match the physical properties of a bulk polymer material, by growing polymer brushes of the same chemical structure as the bulk polymer. This method, as mentioned, is not limited to adhesive interfaces and finds use in the field of hybrid nanocomposite materials as well. Matching the surface chemistry of zirconia NP fillers with a PMMA matrix was utilized by Hu et al [46]. Improved interactions between the filler and matrix resulted in improved mechanical properties, such as increased surface hardness, while largely maintaining other attractive properties of PMMA such as optical transmittance. At the company RadiSurf ApS, the very same concept is utilized to bond bulk PS to SS parts by growing PS brushes from the surfaces, before solvent welding to bulk PS (Table 1). Pull-out testing results in tensile strength of 11 ± 1 MPa and fully cohesive failure (Table 2). 

Even between dissimilar polymer structures, polymer–polymer interactions such as hydrogen bonds and other electrostatic attractions may contribute to a lowering of the enthalpy of mixing, resulting in vastly enhanced interaction and adhesion in the interface. Haase et al. showed the dispersion of silica NPs in a polycarbonate (PC) matrix [47]. This was achieved after growing poly(styrene-co-acrylonitrile) from the particles, a polymer composition known to be miscible with PC in the bulk. Moreover, it is well known that blends of e.g., PMMA and PC or acrylonitrile butadiene styrene (ABS) are miscible [48,49].

Table 2 showcases several examples from the laboratories at RadiSurf ApS where stainless steel or aluminum (Alu 1050) are coated with PMMA brushes and overmolded or heat pressed with either PMMA, PC, or ABS. Lap shear or pull-out tests show a high adhesive strength and cohesive failure for all the material combinations, most likely due to the entanglement of the PMMA brushes into the three thermoplastics. These examples show how favorable physical interactions contribute strongly to adhesion even between dissimilar polymers and demonstrate how thermoplastics and metals can be joined through direct overmolding or welding techniques commonly used in industry via only a thin thermostable polymer brush interphase. Hence, this method of joining dissimilar materials may find applications in industries such as automotive, aerospace, medico, and electronics, where e.g., metal–plastic joints are found in countless products and components. 

In the case where physical interactions between polymer brushes and a bulk polymer matrix cannot be achieved due to unfavorable mixing, adhesion based on covalent bond formation is another option for polymer brush-based adhesion. It is often necessary to design polymer brushes with chemical handles which may react with the bulk polymer. This approach has been successfully used for the incorporation of filler particles with otherwise incompatible materials. Tao et al. grafted PGMA brushes to TiO_2_ NPs to introduce reactive handles, in this case pendant epoxide groups, and enable the reaction with an epoxy resin during curing [31]. At high polymer brush graft densities, high weight percentages of TiO_2_ NPs were dispersed in the epoxy resin, whereas for low graft densities, the filler agglomerated. This research demonstrates the compatibilization of two dissimilar materials through the formation of chemical cross links, and the strategy may be directly applicable for bonding dissimilar materials. RadiSurf has likewise utilized PGMA brushes grown from initiator-modified SS and aluminum to improve the bond strength to epoxy resins (Table 3). Importantly, the lap shear strength increased by 10% and 65% compared to blank, non-functionalized stainless steel and aluminum substrates, respectively. 

Highly nonpolar bulk polymers, such as ethylene propylene diene M-class (EPDM) rubber, largely prevents the interpenetration and entanglement of most polymer brushes. This necessitates the introduction of chemical handles on the polymer brushes to enable strong bonding to the rubber. While alkenes may serve as such handles and react with EPDM during the vulcanization process, alkene-functionalized monomers are not readily compatible with a radical polymerization, and hence such polymer brushes cannot be directly synthesized. In a study by Buhl et al., double bonds were introduced to PGMA brushes on SS in a post-polymerization modification by reaction with allylamine or diallylamine (Table 1) [50]. The immobilized alkene groups facilitated the reaction between the polymer brushes and EPDM rubber during the overmolding and vulcanization process (170 °C for 12 min) and provided strong adhesion between the SS substrates and EPDM. In this case, peel testing was used to determine the strength of the adhesion between the rubber and the SS.

The peel strength of the interface consisting of a nanometer-thin allylamine-modified PGMA brush obtained the same high peel strength as a micrometer-thick layer of a commercial bonding agent, with a full cohesive fracture, i.e., the locus of fracture was in the bulk rubber. However, the PGMA brushes modified with diallylamine showed a lower peel strength compared to the commercial solution and did not obtain a full cohesive fracture. This difference in mechanical performance is attributed to the primary amine (allylamine), which may react with two epoxy groups during the post-polymerization reaction. This forms a cross-linked polymer brush network when incorporated into the PGMA brush, while the secondary amine (diallylamine) cannot facilitate cross-linking in the brush (Table 1). Thus, the process zone in a peel experiment may be increased in a cross-linked brush, distributing the peel force on a larger surface area, and thereby increasing the apparent peel strength of the interface. 

**Figure 2:**
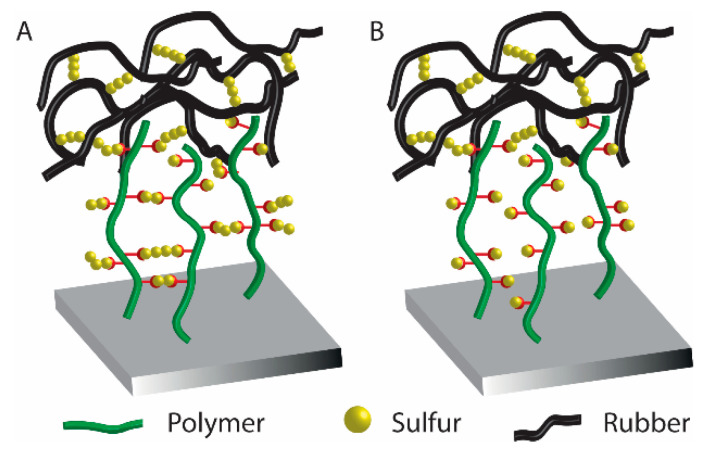
Illustration of (**A**) sulfide cross-linked polymer brushes and (**B**) uncross-linked thiol-containing polymer brushes both with C–S bonds to EPDM rubber, adapted from Buhl et al. [43].

In a recent associated work by Buhl et al., a post-polymerization modification of PGMA brushes with sodium polysulfides was exploited to form sulfide cross-linked polymer brushes, which during overmolding and vulcanization with sulfur-cured EPDM rubber, reacted and formed cross-links (Figure 2) [43]. Notably, the post-polymerization modification was extremely fast (<30 s) and was conducted in an aqueous solution. It was found that the sulfide cross-linked brush showed the same high peel strength as obtained by the commercial bonding agent. Interestingly, the brushes exhibited a much lower adhesive performance, if the polysulfide bonds in the modified polymer brushes were reduced to thiols prior to the overmolding. Again, the difference in adhesive strength was attributed to the fact that the process zone in a peel experiment may be increased in cross-linked brushes, distributing the peel force on a larger surface area.

This post-polymerization modification, carried out in <30 s in non-toxic aqueous/alcohol solvents, makes this procedure highly industrially relevant. Sulfur-based adhesives are most often not allowed in applications for drinking water and food production lines, which makes the polymer brush-based adhesion technology particularly relevant for such applications. Polymer brushes are chemically linked in one end to the base material, providing a very high stability in the adhesive interphase with no risk of chemical leaching. In fact, the cytotoxicity tests performed by Pacific BioLabs (Pacific BioLabs SOP 15B-10, rev. 1I.00, Cytotoxicity—Elution Test (USP Method)) for Aarhus University and RadiSurf ApS in a joint collaboration, has demonstrated that both the PGMA brushes and the sulfide modified system are non-toxic. Briefly, the SS samples coated with PGMA or sulfide-modified PGMA brushes were incubated in an extraction medium, which after 24 h was removed and used as an incubation medium for a monolayer of L-929 mouse fibroblast cells (ATCC Cell Line CCL1, NCTC Clone 929) for 48 h. The condition of the cells was then inspected using light microscopy and found to show either “none” or a “slight” reactivity towards the extraction medium. Both grades of reactivity meet the test requirements of USP 41 <87>, rev. 05/2018. (USP 41 <87>, rev. 05/2018, Biological Reactivity Tests, In Vitro. Compliance Statement: This study was performed for non-regulatory purposes and was not intended to comply with United States Food and Drug Administration (FDA) Good Manufacturing Practice Regulations (cGMP), Title 21 of the U.S. Code of Federal Regulations, Parts 210 and 211 or Good Laboratory Studies (GLP), Title 21 Code of Federal Regulations Part 58.)

In medico and applications for food and beverages, the stability and non-toxicity of adhesive layers are of paramount importance. Poly(dimethylsiloxane) (PDMS) is used in many high-end products, and a polymer brush design for the improved adhesion between titanium substrates and PDMS was developed by Nielsen et al [51]. Poly(2-hydroxyethyl methacrylate) (PHEMA) brushes were grown by activators regenerated by electron transfer (ARGET) ATRP from titanium substrates. A post-polymerization modification of the hydroxyl sidechains of the PHEMA brushes using chlorodimethylsilane served a two-fold purpose. First, the introduced hydrosilane groups acted as a chemical handle which may react with the PDMS during the curing and form covalent bonds to the PDMS, and second, they served to mimic the structure of PDMS, enhancing the physical interactions (Table 1). Adhesion tests showed cohesive failure, i.e., the interfacial strength (~250 N), and outperformed the strength of the bulk material.

The importance of chemical bonding in this system was highlighted by replacing chlorodimethylsilane with chlorotrimethylsilane to post-modify the PHEMA brushes (Table 1). In essence, the hydrogen atom in the hydrosilane group was replaced with a methyl group to eliminate the chemical handle available for creating chemical bonds to the vinyl groups during the curing process of PDMS. Adhesion tests showed complete adhesive failure, although the interfacial strength was measured to be ~190 N. This suggested that good adhesion based on physical interactions had occurred, but not to the same level as given by the chemical crosslinks to PDMS. It was concluded that for PHEMA brushes treated with chlorodimethylsilane, entanglement and covalent bonds act in synergy to ensure strong and reliable bonding of modified titanium to PDMS.

These examples highlight that inventive design of surface chemistry using polymer brushes can enable strong bonding of dissimilar materials by means of both covalent bonding, physical interactions, and entanglements between surface-tethered polymer brushes and bulk polymers. In the pursuit of new, environmentally friendly, and smart bonding systems, we believe that polymer brushes represent a very promising nanotechnology for joining materials in the future. The nanoscale bonding system may be designed for hundreds of different applications and applied on a plethora of substrates. Importantly, for the systems merely relying on entanglement for bonding (Table 1), the adhesion can be reversed by melting or dissolving the polymers just in the interphase (effectively reversing the welding). This mode of reversible adhesion (on demand debonding) allows materials to enter the circular economy and be reused or recycled. Hence, this novel adhesion technology based on polymer brushes is ideal for one of the main components of the European Green Deal [52].

## 4. Progress in Synthesis of Polymer Brushes

Polymer brushes represent a highly promising concept for a large variety of applications, including, amongst others, the formation of strong joints between dissimilar materials as discussed above. However, the extensive research in the field has still not led to the first large scale industrial production of polymer brush-functionalized materials. The scale-up process of SI-ATRP going from laboratory scale (<10 cm^2^ substrates) to industrial scale (~0.1–100 m^2^ substrates) has proven challenging and is a key obstacle for the industrial implementation of polymer brushes. The main issues with SI-ATRP are its oxygen sensitivity, long polymerization times, and the use of hazardous chemicals. To overcome this and go from research laboratory to industry at scale, large investments and pervasive research and engineering are required.

**Figure 3:**
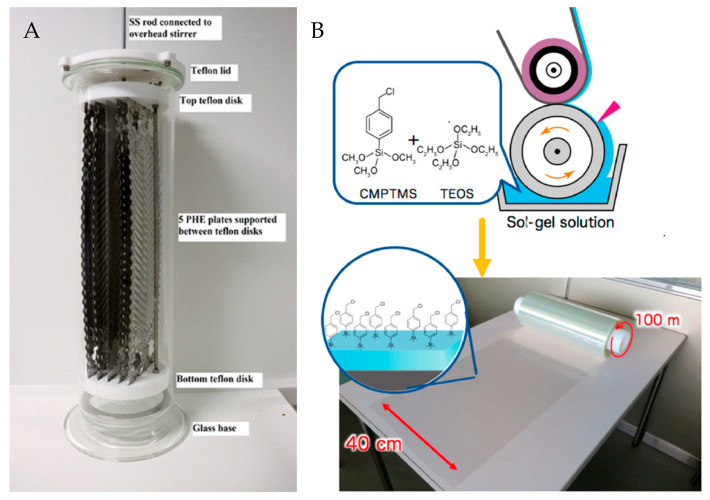
(**A**) Picture of the polymerization setup for the coating of commercial PHE plates. The PHE plates are supported by two teflon disks in a 10 L glass container with an overhead stirring bar, reprinted with permission from Friis et al. [53]. Copyright 2019 Elsevier. (**B**) Illustration of a roll-to-roll coating process on poly(ethylene terephthalate) (PET) film, reprinted with permission from Sato et al. [54]. copyright 2018 ACS.

Few examples exist in the literature where general procedures for the preparation and polymerization of brushes are scaled. In a recent study, poly(oligo(ethylene glycol) methacrylate) (POEGMA) brushes were grafted from commercial plate heat exchanger (PHE) plates (43 cm × 12.5 cm) with chevron grooves [53]. The PHE plates were modified by the direct scaling of the laboratory procedure to an ATRP pilot-scale setup. This meant that coating four PHE plates with POEGMA brushes required 3 L of monomer, 0.14 kg of 2,2′–bipyridyl, 25 g CuBr_2_, and 29 g CuBr in a 10 L glass container (Figure 3A). The plates were grafted with dense, homogenous thin layers of POEGMA brushes without compromising the heat transfer of the PHE plates. The POEGMA efficiently decreased the CaCO_3_ fouling during the operation in water, resulting in an improved lifetime of the heat exchangers.

At the same time, this case demonstrates the issue with the direct scaling of laboratory procedures, where high amounts of reactants are required to form a nano-scale polymer film resulting in a staggering cost per treated plate. Besides, more than 99.9% of the monomer is wasted and disposed as the reaction solution cannot be reused due to the deactivation of the catalyst after exposure to oxygen. Likewise, the polymerization is oxygen sensitive, requiring oxygen-free conditions, which is also not compatible with many industrial settings. 

More promising strategies involving Cu^0^-mediated SI-ATRP reactions have been shown to efficiently functionalize ~30 cm^2^ wafers with a variety of polymer brushes [55]. Here, a 1 µL cm^−2^ polymerization solution was used to afford, in the case of PMMA, ~36 nm-thick polymer brushes in 60 min. A flat copper plate on top of an initiator-functionalized substrate acts as the source of the catalyst, but at the same time also controls the exposure of oxygen during the polymerization. Hence, in this protected sandwich configuration, the reaction is oxygen tolerant, highly versatile regarding the types of monomer and substrate material, and environmentally friendly [55,56]. The work represents a technologically relevant method that is, however, limited to flat substrates and may not be applicable for products with different geometries. Furthermore, the relatively long polymerization time means that the method may not be suited for production lines with high output numbers. 

Work by Dunderdale et al. represents another example of up-scaled SI-ATRP reactions for large-scale polymer brush synthesis under ambient conditions [57]. They present a “paint-on”-ATRP process, where aqueous solutions are applied directly to large-scale initiator-functionalized substrates (10 × 30 cm^2^) in air. An adsorbent paper is placed on the polymerization solution-wetted substrate to prevent reagent evaporation and allow a polymerization time of 90 min. While this “paint-on” approach is promising, the polymerization time is long compared to the timespan of large industrial processing lines. Nevertheless, the paint-on method represents the most relevant process industrially, where large surfaces with different geometries may be polymer brush-functionalized. Furthermore, in a recent study by Sato et al., they demonstrated a roll-to-roll coating method using a low-concentration sol–gel solution to functionalize large poly(ethylene terephthalate) (PET) films (0.4 × 100 m^2^) with an ATRP initiator, at a coating speed of 5 m min^−1^ (Figure 3B) [54]. Subsequently, “paint-on”-ATRP was demonstrated by spreading a 0.02 mL cm^-2^ polymerization solution on 40 × 40 cm^2^ initiator-functionalized PET films, with another film placed on top, in a sandwich structure. After 30 min polymerization using 2-(diethylamino)ethyl methacrylate as monomer, a dry film thickness of ~56 nm was obtained. Similar to the cases described above, the top film may here serve both to control the oxygen exposure and at the same time prevent reagent evaporation.

From an industrial point of view, dip-coating represents another method that may efficiently be added to a process line, while being compatible with most substrate geometries. Dip-coating ensures a uniform, homogeneous layer of polymer films. RadiSurf recently published a patent, where a highly oxygen-tolerant ATRP-related process enables upscaling of the synthesis of polymer brushes [58]. The oxygen tolerance is facilitated by employing an oxygen scavenger, which at the same time activates the catalyst for the polymerization. The process is versatile, allowing the synthesis of a broad range of polymer brush coatings from highly hydrophobic poly(pentafluorostyrene) to hydrophilic poly(2-hydroxyethyl methacrylate) under ambient atmosphere and processing conditions. Furthermore, the polymerization is environmentally friendly, using low monomer concentrations (1−10 v/v%) and only water/alcohol-based solvents. Because of this, along with polymerization times of only 5−10 min, this allows RadiSurf to grow polymer brushes in an industrially relevant framework.

**Figure 4:**
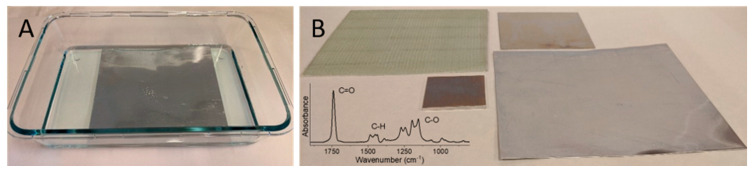
(**A**) Surface-initiated atom transfer radical polymerization (SI-ATRP)-related process in an open polymerization bath. (**B**) Examples of the parts coated with PMMA brushes, including the upper left plate (15 × 15 cm^2^ glass fiber epoxy composite), upper right plate (10 × 10 cm^2^ stainless steel sheet), lower left plate (5 × 5 cm^2^ aluminum sheet), and the lower right plate (18 × 18 cm^2^ aluminum foil). Lower left corner shows the infrared reflectance absorbance spectroscopy of the PMMA brushes on the SS sheet.

Figure 4 shows how PMMA brushes of ~60 nm (dry film thickness determined by ellipsometry) are grown in this manner from initiator-functionalized SS plates in completely open and exposed polymerization baths with no prior oxygen removal. A selection of substrate materials, e.g., SS, aluminum, and glass fiber epoxy composite, compatible with the polymerization, is also shown. Figure 4B (lower left corner) shows a infrared reflectance absorbance spectrum of the PMMA brushes on SS with peaks assigned to C=O bond stretch from an unconjugated ester (1740 cm^−1^), C–H deformations (1490–1440 cm^−1^), and C–O stretch (1280–1050 cm^−1^). Importantly, a recent test of the system by RadiSurf, suggests that the polymerization solution may be reused. This enables higher volumes of parts to be coated in a batch-wise process using one solution only. (Note that the polymer brush coatings were made using the patented procedure by RadiSurf ApS, and thus do not represent novel scientific findings. They are included here only for the purpose of illustrating the progress in upscaling polymer brush synthesis using ATRP-related processes. To support the formation of PMMA brushes using the patented procedure, we provide infrared reflection absorption spectroscopy data from the stainless steel substrate).

To summarize, important steps have in recent years been taken towards scaling up and implementing the process to industrially modify substrates with polymer brushes. The two most critical contributions in this respect are the movement towards oxygen-tolerant polymerization processes and low polymerization times, which in our opinion will be the main drivers for transferring polymer brush synthesis from university laboratories to industrial production lines. The demonstrations of polymer brushes synthesized in both a “paint-on” fashion and efficient dip-coating methods, highlight the maturation of the technology and readiness for scale-up. Hence, polymer brush technology is at a stage to demonstrate its use in real-life application, either as an adhesion promoter discussed in this review or used in the numerous other polymer brush applications.

## 5. Closing Remarks

Amongst all the promising applications of polymer brushes that have been demonstrated at lab scale over the past two decades, polymer brushes as an adhesion promoter hold a very high potential to be scaled up and implemented industrially. A range of dissimilar materials is bonded using polymer brush-based adhesion and shows similar or better performance than the existing commercial bonding solutions, while at the same time demonstrating a low environmental impact and toxicity. The broad scope of materials that may be joined, combined with recent advances in the scale up of polymer brush synthesis, makes the technology extremely interesting for future applications in high-performance products. From our point of view, polymer brush-functionalized materials are at the frontier of adhesion technology.

## Figures and Tables

**Table 1:** Chemical structures of the polymer brushes, post-polymerization reactants, modified brush structure, and the adherends/matrixes discussed in this review.

Polymer Brush	Post-Polymerization Reactant	Modified Brush Structure	Adherend/Bulk Matrix
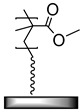 PMMA *^a^*	-	-	PMMA
	-	-	ABS *^b^*
	-	-	PC *^c^*
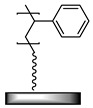 PS *^d^*	-	-	PS
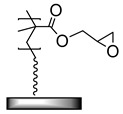 PGMA *^e^*	-	-	Epoxy
	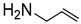	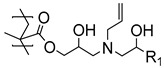	EPDM(p) *^f^*
	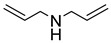	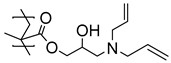	EPDM(p)
	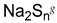	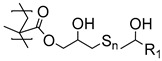	EPDM(s) *^h^*
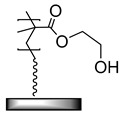 PHEMA *^i^*	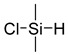	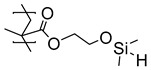	PDMS *^j^*
	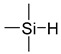	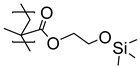	PDMS

*^a^* Poly(methyl methacrylate) (PMMA), *^b^* acrylonitrile-butadiene-styrene (ABS), *^c^* polycarbonate (PC), *^d^* polystyrene (PS), *^e^* poly(glycidyl methacrylate) (PGMA), *^f^* Ethylene–propylene-diene M-class rubber peroxide cured (EPDM(p)), *^g^* n = polysulfuration index, *^h^* ethylene–propylene-diene M-class rubber sulfur cured (EPDM(s)), *^i^* poly(hydroxyl-ethyl methacrylate) (PHEMA), *^j^* Poly(dimethylsiloxane) (PDMS). The R_1_ groups signifies a neighboring polymer chain.

**Table 2:** Various material combinations with bonding relying on the entanglement of polymer brushes into bulk polymers *^a^*.

Substrate	Monomer	Adherend	Strength (MPa)	Failure mode	Test Mode *^b^*
SS *^c^*	MMA *^d^*	PMMA	35 ± 5	Cohesive	Lap shear
Alu 1050	MMA	PMMA	22 ± 3	Cohesive	Lap shear
SS	MMA	PC	29 ± 3	Cohesive	Lap shear
SS	MMA	ABS	17 ± 3	Cohesive	Pull out
SS	Styrene	PS	11 ± 1	Cohesive	Pull out

*^a^* All sample preparation and tensile tests were carried out in the laboratories of RadiSurf ApS. *^b^* Lap shear testing: single lap shear joints were made by heat pressing a polymer foil in between two 50 × 10 × 2 mm^3^ metal lap shear specimens with 10 × 10 mm^2^ overlap. Pull-out test specimens were made by overmolding or solvent welding 10 mm of one end of an 80 mm, Ø 5 mm metal rod with a polymer melt or polymer part. *^c^* Stainless steel (SS) and *^d^* methyl methacrylate (MMA).

**Table 3:** Various material combinations with bonding relying on the cross-linking of polymer brushes with bulk polymers.

Substrate	Monomer	Adherend	Lap Shear Strength (MPa)	Adhesive Force (N)	Peel Energy (N mm^−1^)	Cohesive/Adhesive
SS	GMA *^a^*	Epoxy *^b^*	26.8 ± 0.1	---	---	Cohesive
Alu	GMA	Epoxy *^c^*	26.0 ± 0.6	---	---	Cohesive
Ti	HEMA *^d^*	PDMS *^e^*	---	250 ± 40	---	Cohesive
Ti	HEMA *^f^*	PDMS	---	190 ± 40	---	Adhesive
SS	GMA *^g^*	EPDM(p) *^h^*	---	---	15.4 ± 1.1	Cohesive
SS	GMA *^i^*	EDPM(s) *^j^*	---	---	21.4 ± 0.8	Cohesive

*^a^* Glycidyl methacrylate (GMA), *^b^* epoxy (Araldite^®^ 2001), *^c^* epoxy (3M DP460), *^d^* hydroxyl-ethyl methacrylate (HEMA) post-polymerization modified with chlorodimethylsilane, *^e^* poly(dimethylsiloxane) (PDMS) (MED-4420), *^f^* post-polymerization modification with chlorotrimethyl-silane, *^g^* post-polymerization modification with allylamine, *^h^* ethylene-propylene-diene M-class rubber cured with peroxides (EPDM(p)), *^i^* post-polymerization modification with polysulfides, and *^j^* EPDM rubber cured with sulfur (EPDM(s)).
